# Replacing fentanyl infusion by enteral methadone decreases weaning time from mechanical ventilation

**DOI:** 10.1186/cc10155

**Published:** 2011-06-22

**Authors:** R Wanzuita, G Westphal, F Pfuetzenreiter, S Ayres, A Cavalcanti, LP Figueiredo

**Affiliations:** 1 Medical School, University of Joinville, Joinville - SC, Brazil

## Background

Patients exposed to long-term infusion or high-dose of opioids may develop physiological dependence and withdrawal symptoms during discontinuation. In mechanically ventilated adult patients, the occurrence of fentanyl withdrawal syndrome has been associated with difficulties in discontinuing ventilatory support and with increased length of stay (LOS).

## Objective

We tested the hypothesis that replacement of fentanyl infusion by enteral methadone decreases weaning time from mechanical ventilation.

## Methods

A prospective, randomized and double-blind study involving patients fulfilling criteria to weaning from mechanical ventilation but under high risk for fentanyl abstinence syndrome (defined as continuous fentanyl for more than 5 days or more than 5 μg/kg/hour during 12 hours). Patients were randomized into two groups, methadone (MET) group and control (CT) group, as follows: at first 24 hours both groups were given 80% of the original dose of fentanyl and received, additionally, in the MET group enteral methadone (10 mg each 6 hours) or enteral placebo in the CT group. After the first 24 hours, the MET group received enteral methadone and intravenous placebo while the CT group received enteral placebo and intravenous fentanyl. In both groups, the blinded intravenous solutions were reduced by 20% of the original dose, every 24 hours. Any abstinence symptoms were treated with a bolus of fentanyl. A Kaplan-Meyer curve was constructed and the Student *t *test was used to compare groups in following criteria: (1) weaning time from MV, (2) days under MV and (3) ICU LOS.

## Results

Of 75 randomized patients, seven were excluded and 68 were analyzed: 37 at MET and 31 in CT. Between the beginning of weaning and extubation, there was a greater probability of anticipation of extubation in the methadone group, but the difference was not significant (hazard ratio = 1.44; 95% CI = 0.81 to 2.56; *P *= 0.21). The effects of treatment on weaning time were time dependent, and we observed that on the fifth day the probability of successful weaning was 2.27 times greater in the MET (*P *vs. 13.28 ± 12.85 days, *P *< 0.004). There was no difference between the two groups with respect to the duration of mechanical ventilation and ICU LOS.

## Conclusion

These data show that replacement of fentanyl infusion by enteral methadone reduces the weaning time from mechanical ventilation.

